# A soft policy instrument with a hard content? An analysis of national agreements in Swedish multi-level healthcare

**DOI:** 10.1108/JHOM-12-2024-0527

**Published:** 2025-06-24

**Authors:** Jessika Wide, David Feltenius

**Affiliations:** Department of Political Science, Umeå University, Umeå, Sweden

**Keywords:** Healthcare, Multi-level system, Governance, Policy instruments, Agreements, Sweden, Decentralization

## Abstract

**Purpose:**

In Sweden, a decentralized unitary state, the 21 self-governing regions are responsible for healthcare. A governing tool in the relations between the central government and the regions is the use of “national agreements”. The literature describes agreements as soft policy instruments, meaning they are voluntary, non-binding and lack detail. Our purpose is to analyse the content of national agreements in Swedish healthcare to explore their characteristics.

**Design/methodology/approach:**

We use document analysis to investigate whether the content of the national agreements in Swedish healthcare is primarily general or specific in terms of stated goals, measures, disbursement and review. The analysis includes eight agreements from 2022.

**Findings:**

The content varies in specificity and detail across agreements in healthcare, some being specific while others are more general and broadly formulated. An important conclusion is that agreements within the same policy area can have different characteristics. Agreements are inherently “soft”, but with potential variations in their application and consequences.

**Originality/value:**

Due to their high level of detail and scope, agreements may pose a challenge to the principle of regional self-government. This could limit the regions’ discretion to set their own priorities and make necessary adjustments, ultimately impacting both efficiency and accountability within healthcare.

## Introduction

The literature has long discussed the balance between central government control and local government discretion in welfare states (Page and Goldsmith, 1987; Blom-Hansen, 1999; Rhodes, 1999; Goldsmith and Page, 2010). An essential issue is the type of policy instruments that the central government applies to steer subnational authorities. In Sweden, the responsibility for planning and providing healthcare is decentralized to 21 regions with self-government (Fredriksson *et al.*, 2014; Blomqvist and Winblad, 2021, 2023, 2024). The regulation of Swedish healthcare by legislation primarily consists of framework laws. The principle of regional self-government is based on democratic assumptions and the belief that regions are better positioned to assess the needs of their own population and design locally adapted systems of healthcare provision. At the same time, universalism is an important value in Swedish welfare, and all citizens should have equal access to high-quality healthcare regardless of where they live (Montin, 2016; Blomqvist and Winblad, 2021).

Accordingly, a variety of additional policy instruments have emerged in the Swedish welfare state during the past decades, such as national guidelines and quality monitoring conducted by central government authorities (Fredriksson *et al.*, 2014; Blomqvist and Winblad, 2021, 2023, 2024). Another example is “national agreements” between the central government and the Swedish Association of Local Authorities and Regions (SALAR), the local government association that represents the regions and advocates their interests (Feltenius, 2016; Feltenius and Wide, 2025). These agreements cover prominent areas in healthcare, such as cancer care, maternity care, waiting times, and integrated care. They outline desirable objectives and activities that fall within the regions’ responsibilities and include financial support in the form of targeted government grants (SALAR, 2022; Statskontoret, 2023). While these agreements are voluntary for the regions, past investigations indicate that all regions choose to “join” (Fredriksson *et al.*, 2012; SNAO, 2014).

Agreements could also occur between subnational units as a tool to enhance collaboration and coordination in healthcare at the meso-level (e.g. Rudkjøbing *et al.*, 2014; Vabo and Burau, 2019). However, in this article, we will explore the national agreements at the macro-level through which the central government aims to steer the regions. In the literature on policy instruments, agreements of this kind are classified as a “soft” policy instrument (Jordan *et al.*, 2005; Fredriksson *et al.*, 2012). This type of policy instrument has several distinctive features, such as being non-binding, non-sanctionable, flexible, and deliberate (Wurzel *et al*., 2013a, b; Blomqvist, 2022). Additionally, it often has vague and aspirational goals, creating discretion for those involved (Karlsson-Vinkhuyzen and Vihma, 2009; Blomqvist, 2022).

It has been noted that the alignment between hard and soft categories of policy instruments “is often quite tenuous” (Zehavi, 2012, p. 245). Therefore, a crucial question arises about how the content of agreements is structured in terms of being either “general” or “specific”. For example, if agreements are highly detailed and performance-based, one might question if they are different from “hard” policy instruments. This is significant because “hard” steering, striving for national equity in healthcare, may be ineffective as it overlooks the differences in conditions between regions and the specific needs of their residents. Furthermore, it could go against the political priorities of the self-governing regions and their organizational preferences in healthcare (Montin, 2016). If so, the agreements challenge the regional self-government and are rather a tool for healthcare recentralization (Saltman, 2008; Fredriksson *et al.*, 2014; Blomqvist and Winblad, 2023, 2024).

We depart from the notion that national agreements in healthcare are *documents*. Documents are significant in public policy and, in fact, we “cannot understand government without understanding documents” (Freeman and Maybin, 2011, p. 156). To fully understand national agreements in Swedish healthcare as a policy instrument, our study aims to systematically analyse the content of the agreements in terms of steering. We ask two questions: Firstly, can the content of the agreements be primarily categorized as “general” or “specific”? Secondly, what implications does this categorization have for agreements being “hard” or “soft”, and ultimately for the discretion of the regions within healthcare? Our empirical investigation is limited to the written content of the documents and does not include the process of development or implementation.

## Theoretical background

### Hard and soft policy instruments

Policy instruments are well explored in previous research. Research on policy instruments has, for example, investigated how governments govern, resulting in various categorizations (e.g. Hood, 1983; Schneider and Ingram, 1990; Vedung, 1998). The categorization of “soft” and “hard” policy instruments is well established. Policy instruments with a high degree of authoritative force, such as regulations, are considered “hard”, while those with a low degree of authoritative force, such as informative instruments, are considered “soft”. However, the possibilities of sanctions are only one of many differences between hard and soft policy instruments. According to Paula Blomqvist (2022), there are at least five dimensions along which differences can be noticed.

In Table 1, an important distinction to consider is whether the policy instruments are binding. Hard policy tools, such as regulations, are legally binding and mandatory, while compliance with soft policy tools is voluntary. Non-compliance with hard policy instruments can lead to sanctions and enforcement issued by courts or agencies, unlike soft policy instruments. Moreover, hard policy instruments have detailed and precise content, limiting the receiver’s discretion, whereas soft policy instruments are vague and adaptable, giving the receiver a greater degree of discretion (Blomqvist, 2022).

**Table 1:** Dimensions of soft and hard policy instruments

Dimension	Hard policy instruments	Soft policy instruments
Binding	Yes	No
Sanctions	Yes	No
Content	Precise	Vague
Form of decision	Fixed	Flexible
Steering logic	Authoritative	Deliberative

**Source(s):** Based on Blomqvist (2022, pp. 285–288)

Another distinction is the form of decision-making, which impacts the level of flexibility. Hard policy instruments are rigid and necessitate parliamentary involvement for decisions and amendments, whereas soft policy instruments are more adaptable as they can be issued and amended by the government alone or by public agencies, without political struggles. Additionally, deliberations play a crucial role in soft policy instruments, as opposed to commands from higher levels in a strict hierarchy of institutions and actors (Blomqvist, 2022).

### Agreements as a soft policy instrument

Previous literature classifies agreements as a soft policy instrument (e.g. Rennings *et al.*, 1997; Jordan *et al.*, 2005; Fredriksson *et al.*, 2014; Blomqvist and Winblad, 2024). Therefore, the different dimensions of soft policy instruments as described above are relevant to consider for agreements.

The literature on agreements as a soft policy instrument is primarily found in the field of environmental policy (Rennings *et al.*, 1997; Brink *et al.*, 1999; Mol *et al.*, 2000; Brink, 2002; Croci, 2005; Pearce and Cooper, 2011). This literature includes various types and definitions of agreements. According to Wurzel *et al.*, (2013b), referencing to a German study, there are more than twelve terms for agreements in the German language, such as negotiated agreements, voluntary agreements, co-regulation, and voluntary accords. Examples of agreements between the central state and subnational authorities can also be found in the literature, for example, in spatial planning in France (Booth, 2009), in social policy in Belgium (Wayenberg, 2006), and in Norwegian eldercare. In Norway, these agreements are described as “soft and process oriented” (Vabo, 2012, p. 122). Still, in the literature, agreements in environmental policy dominate.

According to data from the mid-1990s, all EU member states have adopted voluntary agreements within environmental policy, but the Netherlands and Germany have been particularly active (Jordan *et al.*, 2003; Wurzel *et al*., 2013a, b). An insight from this literature is that agreements, when considering their content, can take many different forms. It has been concluded that voluntary agreements do not constitute a “uniform instrument” (Gebers, 1998, p. 91). Firstly, complementary instruments, such as government grants and taxes, can be attached to agreements to various extents (Brink *et al.*, 1999). Secondly, some agreements are detailed and very specific in their content, while others are less so. In an overview of environmental agreements in six EU countries, attention was paid to their level of detail, especially concerning the targets’ degree of precision, reporting requirements, and compliance incentives (Brink *et al.*, 1999).

### Harder soft governance

Based on previous literature, it is expected that the level of detail and governing impact of agreements will vary. This contradicts the common perception of soft policy instruments, which are typically associated with vague content and goals (Karlsson-Vinkhuyzen and Vihma, 2009; Blomqvist, 2022). This inconsistency has also been recognized in the broader literature on soft and hard policy instruments. It has been proposed that the connection between hard and soft categories and actual policy instruments is “often quite tenuous” (Zehavi, 2012, p. 245).

This theme is further explored in research on the development of soft policy instruments and their incorporation of harder elements. The integration of harder elements into soft policy tools is referred to as “harder soft governance”, HSG (de la Porte and Stiller, 2020; Knodt *et al.*, 2020; Knodt and Schoenefeld, 2020; Schoenefeld and Jordan, 2020). According to Michele Knodt and Jonas J. Schoenefeld, HSG tends to arise in situations where traditional hard governance, such as laws, is not feasible while traditional mechanisms of soft governance are insufficient (Knodt and Schoenefeld, 2020).

The presence of HSG can be identified through various indicators, including *precision* in steering documents, such as agreements (Knodt and Schoenefeld, 2020). Texts can be written with *high* precision, for example, using “determinate rules” with clear and unambiguously stated objectives and means, or with *low* precision in terms of “vague and general” formulations that allow discretion for the actors being targeted (Abbott *et al.*, 2000). In this context, HSG is evident through the specificity of the targets, such as an exact percentage of emissions reductions (Knodt and Schoenefeld, 2020). Therefore, soft governance becomes harder through a more precise formulation (Knodt *et al.*, 2020). In addition to this dimension of precision, HSG is also characterized by opportunities for blaming and shaming procedures, connections with other policy fields where sanctions are possible, the involvement of third-party actors equipped with enforcement instruments, and justifications that describe the extent to which receivers must explain why and how they react to a recommendation (Knodt and Schoenefeld, 2020).

### Summary

We argue that it is important to acknowledge the potential for variety within soft policy instruments in terms of steering and precision. This article will focus on one aspect of agreements as a soft policy instrument, namely *the content*. If the agreements are detailed with clear and unambiguously stated objectives and means, they will have implications for the regions’ self-government and discretion in healthcare. The rationale behind using soft policy instruments in central–regional government relations is that they allow the regions to make decisions in accordance with regional preferences, conditions, and needs of the residents. Therefore, soft policy instruments fit well with the principle of regional self-government, which is established in the Swedish constitution. However, this rationale no longer applies if the agreements are specific and very detailed. For example, this will result in a reduction of the regions’ discretion to define what constitutes good healthcare and how to best deliver it based on local conditions, preferences, and priorities (Fredriksson *et al.*, 2014, p. 7).

## Setting the context

In the literature, Sweden is often referred to as a decentralized unitary state (Lidström, 2012). This means that regulatory and supervisory powers are centralized at the national level, while the 21 regions are responsible for planning, funding, and providing high-quality healthcare to their residents. The regions have the authority to levy taxes and are governed by politicians directly elected in general elections. The eight national parties also dominate in the regions, although there are some local parties present (Blomqvist and Winblad, 2021).

The national agreements in healthcare are negotiated between the Ministry of Health and Social Affairs (MoHSA), on behalf of the central government, and the Swedish Association of Local Authorities and Regions (SALAR) (Feltenius and Wide, 2025). SALAR is a politically run organization, with all of Sweden’s 290 municipalities and 21 regions as members. Political positions within SALAR, for example, in the board and the preparatory bodies, are filled by politicians from municipalities and regions nominated by the political parties centrally. The civil servants employed by SALAR are organized in different departments based on areas of expertise. SALAR’s aim is to represent the interests of the regions and municipalities, acting as a lobbying organization towards the central government and as a mediator or negotiator between different levels of the political system (Feltenius, 2016).

In the early 1970s, the then Federation of County Councils (FCC) and the central government began formal negotiations on healthcare and its costs (Garpenby, 1995). In 1985, they reached an agreement known as “the Dagmar Agreement”, which was more comprehensive than current healthcare agreements as it covered various areas of development (Anell and Svarvar, 1999). Subsequently, other agreements were also reached, such as the “Sickness Bill Billion” in 2006 (SNAO, 2014). During 2019–2022, the number of agreements in operation on healthcare was 10–12 per year (Statskontoret, 2020, 2021, 2022, 2023) [1]. These agreements consist of rather extensive documents containing, for example, objectives and means, making them a suitable case for examining how agreements in a decentralized, multi-level healthcare system like Sweden is characterized.

## Methods and materials

### Selection of empirical materials

We limited our study to agreements in healthcare that were in operation in 2022, a year that did not differ from previous years in either the number of agreements or their themes. We include eight agreements in healthcare in operation 2022, with four supplements and one amendment (Table 2). These are also presented in Appendix [2]. The agreements are in the public domain and are available in the archive on the central government’s website, thereby guaranteed as authentic [3].

**Table 2:** National agreements for 2022 included in the analysis

Cancer care
Civil Defence (incl. supplement)
Covid-19 Testing
Covid-19 Vaccination
Increased Accessibility (incl. supplement)
Integrated Care
Mental Health (incl. supplement)
Women’s Health (incl. supplement and amendment)

**Note(s):** Full names of the agreements are listed in the Appendix

**Source(s):** Authors’ own work

### Analytical method and framework

We utilize qualitative document analysis, a suitable method for systematically reviewing and evaluating documents. This iterative process involves three steps: *skimming* (superficial examination), *reading* (thorough examination), and *interpretation* (Bowen, 2009).

During the first step, we skimmed the agreements to gain an overview of their scope, focus, and nature. In the second step, we carefully read the agreements multiple times. Given the length of the agreements (10–52 pages), a key task was identifying relevant sections. Sections pertaining to tasks for the central state, municipalities, and SALAR were excluded as they fell outside the scope of our article. We then used an inductive approach to identify themes that could be used as categories for analysis (Bowen, 2009). We searched for themes that were significant and relevant to our interest in agreements as a policy instrument that could potentially steer the regions. The sections that focused on the regions had a similar structure, and we identified four themes in the presentation:

1. the objectives that the regions should achieve according to the agreement (*goals*),
2. the activities that the regions should undertake according to the agreement (*measures*),
3. requirements for the allocation of funds to the regions (*disbursement*), and
4. requirements for the follow-up of the regions (*review*).

These themes guided us in organizing the material before the third and final step – interpretation. Initially, we focused on how the agreements articulated goals and measures for the regions. Subsequently, we examined the financial aspects and accounting requirements stipulated in the agreements.

To categorize and interpret the content of the documents, we developed an analytical framework through the interaction between previous research and our readings of the documents. We assume that the content of the agreements may vary, from general and broad formulations to specific and detailed ones (see Vabo and Burau, 2019). We adopt a dichotomous typology in our analysis since we want to evaluate whether the agreements’ goals, measures, disbursements, and reviews are primarily *general* or *specific* (Table 3). A dichotomous typology may be criticized for hiding variation along a scale, but the advantage of a simplified typology is its clarity in a deductive analysis. Thus, our dichotomous typology, as in previous research, allows us to make general conclusions about national agreements as a governing tool (Blomqvist, 2022) and their implications for regions’ self-government in healthcare (see Fredriksson *et al.*, 2014). For instance, an agreement with specific measurable goals and required measures likely allows less regional discretion, whereas an agreement with qualitative goals and examples of measures may allow greater discretion for the regions. Similarly, a performance-based agreement linking economic grants to measurable regional performance signifies limited discretion, while grants based on population size, allocated in advance, allow for a larger discretion.

**Table 3:** Analytical framework for investigating the agreements

	General	Specific
Goals	Comprehensive and qualitative goals	Measurable and quantitative goals
Measures	The agreement states examples and mentions regional adjustments	The agreement requires specific efforts with a high level of detail
Disbursement	Standard amount or amount based on population size, allocated in advance	Amount based on performance or goal achievement, allocated afterwards
Review	Description of efforts, activities, and results in a report	Reporting of performance in a database

**Source(s):** Authors’ own work

Firstly, *the goals* in an agreement can be either general or specific. *General goals* are comprehensive, qualitative formulations, such as developing new routines or carrying out an educational effort. *Specific goals*, on the other hand, include measurable, quantitative goals, for example, percentage improvements in a particular area. Furthermore, formulations such as “shall” and “must” are used rather than “should” and “can”. Regarding general goals, the regions themselves may decide on local adaptations, considering factors such as population density, economic conditions, organization of care or specific needs. Similarly, *the measures* in an agreement can also be either general or specific. *Specific measures* include activities that the agreement requires to be carried out, while *general measures* consist of examples.

Finally, the agreement’s model for *disbursement and review* can be based on either trust (general) or performance (specific). In a *trust-based* model, the regions choose their means to reach the goals of the agreement, and the review consists of a description of how the grants, which are allocated in advance, have been used. In a *performance-based* model, the regions must instead achieve specific, measurable goals to be allocated grants, and this is distributed after the regions have reported their achievements, most often in a database.

We first identified the relevant sections of the selected agreements for the four themes. We then used our analytical framework (Table 3) to categorize the sections as either “general” or “specific”. In the categorization, we strived for both objectivity and sensitivity (Bowen, 2009, p. 32). To ensure *objectivity*, both authors separately read each document several times and made a categorization. These categorizations were then compared to make sure they were the same. To handle the issue of *sensitivity* to the materials, we carefully discussed how to deal with ambiguities in an agreement, for example, elements of both general and specific goals in the same sections. We chose to categorize the agreements based on the dominant element since no agreement had equal parts of general and specific elements [4]. Instead, either there are only elements of one, or it is the case that one clearly dominates. Based on this, we consider that our categorization of the agreements’ themes is credible.

## Results

In Table 4, we present the results of our empirical analysis, structured according to the four themes of goals, measures, disbursements, and review.

**Table 4:** Results of the empirical analysis of the agreements in Swedish healthcare (2022)

Agreement	Goals	Measures	Disbursements	Review
Cancer Care	Specific	Specific	General/Specific	Specific
Civil Defence	General	General	General	General
Covid-19 Tracking	Specific	Specific	Specific	Specific
Covid-19 Vaccination	Specific	Specific	Specific	Specific
Increased Accessibility	Specific	General	Specific	Specific
Integrated Care	General	General	General	General
Mental Health	General	General	General	General
Women’s Health	General	General	General	General

**Source(s):** Authors’ own work

### Goals

Table 4 shows that four agreements have specific goals, while the remaining four have general goals.

The goal of “Cancer Care” mainly concerns the regions’ work with standardized cancer patient pathways (CPP): 70% of new cancer cases should be investigated in a CPP (inclusion target) and within the set time limits (lead time target) in 80% of the cases (MoHSA/SALAR, 2021d). The targets of “Increased Accessibility” are measurable too, namely a shortening of the regions’ waiting times and a high fulfilment of the guarantee of a limited waiting period for people seeking care. Nine different care interventions are included, and the targets are described in detail, for example, “an improvement by at least 5% units” (MoHSA/SALAR, 2022b, p. 16). A minor goal is for the regions to improve their strategic work with accessibility. The goals of these two agreements consist of specific, quantitative targets and improved performance: The regions *must*, for example, reach the inclusion target in cancer care and shorten the waiting time in healthcare. There is no room for regional interpretation or adjustments.

The main goal of “Covid-19 Vaccination” is “as high and even vaccination coverage as possible in the population” (MoHSA/SALAR, 2021a, p. 7). It is also described in more detail that “a vaccination coverage of at least 80% at the national level must be achieved by week 3 among the population aged 50 and over” (MoHSA/SALAR, 2021a, p. 8). The goal of “Covid-19 Testing” is about the regions having appropriate testing and infection tracking with high cost-efficiency (MoHSA/SALAR, 2021e, p. 4). These goals are all based on recommendations from the Public Health Agency of Sweden, and there is no room for interpretation or regional adaptation.

The goals of the remaining four agreements are qualitative and indicate ambitions and directions. For example, a goal of “Women’s health” is that all women should feel safe and receive safe care at the right time regarding pregnancy, childbirth, and aftercare. Yet certain ambitions that “the government and SALAR agree to work for” are written in more detail and at the operational level, for example: “The staffing is such that one midwife or other relevant competence is available for the woman giving birth during the delivery” (MoHSA/SALAR, 2019, p. 9). The goal of “Mental health” is to strengthen and improve the ongoing work in the regions and municipalities with mental health and suicide prevention, based on “jointly identified development needs”. The care should be knowledge-based, equal, resource-efficient, and of high quality (MoHSA/SALAR, 2021c).

The goal of “Civil Defence” is to increase the robustness of healthcare in crises and war. It includes the regions’ work with preparedness planning and security of supply in healthcare in cooperation with other authorities (MoHSA/SALAR, 2021b). The goal of “Integrated Care” is to support the development of the healthcare system towards providing good quality, local healthcare. It should be based on the needs and conditions of the regions, aiming to increase quality and patient participation, improve accessibility, and ensure resource efficiency (MoHSA/SALAR, 2022a).

The goals of these four agreements are, as already mentioned, *general*. For example, care interventions should be “improved”, “developed”, and “strengthened”, and ambitions should be “worked for”, but it is not further specified what this exactly means or how it can be evaluated. We interpret this as the regions deciding this themselves, based on their specific conditions and needs.

### Measures

The agreements also state measures—or assignments. These could either be general, mainly consisting of examples, or described in detail, including specific activities that the regions are required to complete.

Both agreements addressing Covid-19 outline specific measures. In the “Covid-19 Vaccination” agreement, a key assignment is to “carry out vaccinations against Covid-19 in line with the goals of this agreement and following the Public Health Agency’s guidelines for vaccination and prioritization of groups” (MoHSA/SALAR, 2021a, p. 11). These guidelines were specific, including order of priority, age groups, and intervals. Additional measures include reporting vaccination data to the Agency and communication efforts. In the “Covid-19 Testing” agreement, a primary measure is “to conduct PCR testing to such an extent that individuals who, according to the guidelines of the Public Health Agency, should be tested for Covid-19 can access free testing” (MoHSA/SALAR, 2021e, p. 6). Another measure is to conduct infection tracing following the Agency’s recommendations. The recommendations from the Agency were specific in this case as well.

The “Cancer Care” agreement outlines specific efforts in the form of standardized cancer patient pathways (CPP) with detailed routines and targets. Additionally, the regions must improve waiting times and use a patient survey to follow up on the CPP (MoHSA/SALAR, 2021d).

The remaining agreements contain general measures, explicitly stating that regions may choose the priorities they consider most appropriate. For example, in “Women’s Health”, it states that regions “choose which measures they consider to be most beneficial in achieving the goals outlined in the agreement” (MoHSA/SALAR, 2019, p. 9). The measures mentioned are general and widely held. This is further supported by examples provided of how the funds “may be used” for, such as “developing the chain of care based on women’s needs” (p. 10) and “initiatives in neonatal care” (p. 12).

In the “Mental Health” agreement, regions are expected to develop regionally adapted action plans focusing on using knowledge-based and preventive approaches to increase accessibility and quality of care. The agreement outlines various specific areas with allocated funds. Among other things, regions must develop patient-centred working methods, strengthen child and youth psychiatry, increase user participation, and reduce waiting times. However, the agreement does not provide a detailed description of what these actions entail (MoHSA/SALAR, 2021c).

The agreement “Civil Defence” outlines several measures that regions must implement to enhance robustness, such as war organisation, coordination and command, planning, education and exercises, and security of supply. Yet the measures are formulated generally, for example: “The region must plan to ensure it can maintain a certain level of ability to provide healthcare during times of war” (MoHSA/SALAR, 2021b, p. 20). In the “Integrated Care” agreement, the regions must implement measures that support the transition to good quality, local healthcare. Funds can be utilized for “general measures that are based on a person-centred approach” and measures to ensure “an adequate supply of competence” (MoHSA/SALAR, 2022a, p. 17). Specific measures include fostering collaboration between regions and municipalities, transitioning patients from hospital care to primary care, optimizing resource utilization in healthcare, and improving accessibility in primary care. Regions are also expected to develop action plans to meet targets for registered healthcare contacts and registered general practitioners in primary care (MoHSA/SALAR, 2022a).

In the “Increased Accessibility” agreement, the description of expected measures is minimal. The only specific measure in the agreement is for the regions to create an action plan to improve accessibility and reduce waiting times, consisting of ten mandatory points. These include developing routines, ensuring the quality of waiting lists, conducting controls and follow-ups, and outlining planned measures if targets are not met (MoHSA/SALAR, 2022b). Our interpretation of the agreement is that the measures outlined are general. It does not specify exactly what actions the regions must take, but rather leaves it up to them to determine the necessary steps to achieve the targets. While this gives the regions some discretion in prioritizing and designing their measures, they are still constrained by the precise nature of the targets.

### Disbursement

Our third theme focuses on the criteria for distributing funds to the regions, specifically the government grants tied to the agreements. These grants can be disbursed either *in advance* or *after* performance has been evaluated.

In two agreements (“Women’s Health” and “Civil Defence”), the Legal, Financial and Administrative Services Agency (LFASA) distributes the funds in advance. The amount distributed to each region is mainly based on population size. In one agreement (“Mental Health”), SALAR distributes the funds in advance. These agreements are examples of trust-based disbursement: The regions get funds in advance to work with certain areas.

The funds for “Cancer Care”, based on population size, are paid to the regions on two occasions: at the beginning of the financial year and after the regions report on performance. To receive the second disbursement, the regions must achieve the inclusion target of 70% and use the patient survey (MoHSA/SALAR, 2021d). Accordingly, it is both performance- and trust-based.

Most of the funds for “Integrated Care” for 2022 are paid in advance by LFASA based on the population size. However, the region must have submitted an interim report for 2021 and meet the agreement’s requirements for each development area. A small portion of the agreement’s funds is performance-based: regions that increase the number of clinical education weeks for nursing students receive additional funds. These funds are distributed retrospectively based on the region’s share of the total increase in clinical education weeks (MoHSA/SALAR, 2022a). Yet it is mainly trust-based.

The funds for “Increased Accessibility” are primarily distributed based on the region’s performance in reducing waiting times. This is done through a complex model that utilizes points and bonuses. If a region fails to meet the requirements to receive 100% of its funds, those funds are transferred to a “bonus pot”. At the end of the year, the bonus pot is distributed among regions based on the number of performance requirements they have achieved. Each achieved performance requirement earns 1 point. The more requirements met, the more points a region receives and the larger their share of the bonus pot. A small portion of the funds is allocated in advance to cover costs associated with regional action plans, based on the population size (MoHSA/SALAR, 2022b). This agreement is mainly performance-based.

The funds for “Covid-19 Testing” primarily consist of a fixed amount per test if the test is conducted in accordance with the guidelines. The disbursement is made to the regions by the Public Health Agency at three-month intervals at the end of each period and is determined by the number of reported tests. Additionally, funds are allocated for ongoing work with infection tracking, based on population size, and are paid by LFASA (MoHSA/SALAR, 2021e, pp. 7–9). The “Covid-19 Vaccination” agreement is similar. It includes a fixed amount per vaccination with higher compensation for individuals receiving their first and second doses. The disbursement is made to the regions by LFASA at three-month intervals at the end of each period, based on the number of reported injections. Funds are also allocated to the regions for ongoing costs, partly based on population size, and are paid by LFASA in advance (MoHSA/SALAR, 2021a, pp. 15–16). We view these two agreements as performance-based.

### Review

Our fourth theme concerns the requirements for the regions’ follow-up. As shown in Table 4, four of the agreements have a specific and detailed review process, while the other cases have a more general one.

Most agreements require regions to deliver a report, describing their achieved goals, efforts, and challenges. For example, in the “Women’s Health” agreement, regions submit annual reports to SALAR. These reports must include an action plan outlining the activities planned for the year and how funds will be allocated. The action plan should encompass all efforts outlined in the agreements. If no measures are taken within a specific area, the region must comment and provide a rationale for this (MoHSA/SALAR, 2019). In the “Civil Defence” agreement, regions report their efforts, results, and challenges, along with a description of the fund’s utilization. This report is submitted to the National Board of Health and Welfare (NBHW) (MoHSA/SALAR, 2021b). In the “Mental Health” agreement, regions complete a report based on a standardized questionnaire detailing activities, results, and fund utilization. These reports are sent to SALAR, which then reports to the Ministry, NBHW, and the Public Health Agency (MoHSA/SALAR, 2021c).

In the “Integrated Care” agreement, the regions detail their efforts, results, and challenges, as well as fund utilization for each development area. The reports are submitted to NBHW. Additionally, the regions report the number of clinical education weeks to NBHW (MoHSA/SALAR, 2022a).

Overall, the review of the agreements presented so far is based on the funds being paid out to the regions in advance, primarily according to population size. The government trusts the regions to deliver the efforts outlined in the agreements. Following this, the regions are required to submit an annual report and action plan, detailing the goals and efforts made. However, in most cases, the agreement does not mention what will happen if any area is omitted, other than some agreements stating that it must be justified by the regions. This suggests that the regions have discretion regarding the prioritization and design of initiatives within the area.

In the remaining agreements, the review is detailed and specific, based on the funds being paid out to the regions after measurable performance. For example, in the “Cancer Care” agreement, the regions must report data on waiting times and how inclusion and lead time goals are met (MoHSA/SALAR, 2021d). In the “Increased Accessibility” agreement, the regions must report “quality-assured waiting time data” to the national waiting time database, administered by SALAR (MoHSA/SALAR, 2022b, p. 22). The regions must also submit their action plans and reports to the Ministry and NBHW. In “Covid-19 Testing”, the regions must report the number of conducted tests, and in “Covid-19 Vaccination”, the number of injections. Additionally, the regions in both agreements on Covid-19 must submit financial reports of funds used for the ongoing costs (MoHSA/SALAR, 2021a, 2021e).

## Concluding discussion

This article has focused on national agreements, which are a governing tool in the relations between the central government and the regions in Swedish healthcare. In the literature, agreements are described as voluntary and non-binding, making them an example of “soft” governance (e.g. Wurzel *et al.*, 2013a). This contrasts with laws, which are mandatory to follow. However, there are other dimensions along which differences exist, such as the level of detail. Hard policy instruments are defined as precise and legally binding, while soft ones are characterized by vague content and goals (Karlsson-Vinkhuyzen and Vihma, 2009; Blomqvist, 2022). Whether this division truly applies is, as we have argued in our article, an empirical question. To draw general conclusions on the actual nature of agreements as a policy instrument, a more in-depth analysis of the content of agreements is necessary. Our article is a step in that direction.

Our first research question was to examine whether the content of national agreements in Swedish healthcare can be primarily categorized as “general” or “specific”. We analysed eight agreements that were in effect during 2022. Our findings show that many agreements had a general and broadly formulated content. For example, a common goal was that the care within a certain area “must be developed and strengthened.” Another common formulation was that the regions could choose the measures that would be of the greatest benefit to achieve the goals. The models for economic compensation and review were often trust-based: grants were given in advance, followed by a report detailing how the funds were utilized, what measures were conducted, and why. However, a couple of agreements had a largely specific content with quantitative targets and performance-based grants.

Our investigation reveals that while the format of agreements in healthcare may be similar, such as the structure of the documents and the actors involved in the decision-making process, the differences in the content of these agreements are extensive. This leads to our second research question. The analysed agreements varied significantly in their level of detail and scope. Some agreements only address specific issues, while others aim for a transformation of the healthcare system. Therefore, an important conclusion is that agreements even within the same policy area can vary significantly in character. This has implications for the perception of agreements as a governing tool: always “soft” by definition, but with a wide range of applications and consequences.

We place our results in a theoretical context where policy instruments such as agreements are classified as “soft” in comparison to, for example, laws, which are mandatory and come “from above”. We want to challenge this assumption by emphasizing that the concepts of hard and soft policy instruments are multi-dimensional (Blomqvist, 2022). Soft policy instruments could be soft on some dimensions but not on others. For instance, some agreements may be soft on most dimensions, while others are characterized by detailed control and strict requirements for economic compensation. In the latter case, we interpret this as an example of “harder soft governance” (HSG) that has primarily been discussed in the field of environmental policy (Knodt and Schoenefeld, 2020). HSG highlights the incorporation of harder dimensions in soft policy instruments, which is, according to our findings, important to consider in the context of healthcare policy as well.

Our results also reveal a scenario that has implications for regional self-government within the Swedish multi-level healthcare system. Initially, there appears to be a seamless alignment between agreements and the principle of regional self-government. Agreements are typically described in the literature as voluntary and assumed to be broad in scope. However, this alignment may be questioned in the case of agreements in Swedish healthcare. It is evident that agreements can be more detailed than laws. Our result supports previous research indicating that soft policy-instruments may limit the regions’ discretion in determining their own political priorities and adjusting the provision and organization of healthcare to regional conditions and needs (Fredriksson *et al.*, 2014; Blomqvist and Winblad, 2024).

The Swedish regions have diverse conditions, needs, organizations, and resources, leading to varying priorities and approaches. These are fundamental aspects of the regional self-government. The agreements challenge the principle of self-government due to their level of detail and inflexibility. This could potentially hinder both efficiency and accountability in healthcare. Moreover, the regions encounter challenges in waiving the agreements, as they pertain to essential areas of care that often garner public attention, including waiting times, cancer care, and maternity care. This raises the question of whether the agreements are genuinely voluntary.

Furthermore, the agreements also have other implications for the regions. Agreements are short-term, often lasting for only a year at a time (Statskontoret, 2023). The expectations, goals, and grants outlined in an agreement for one year may not necessarily apply to the following years. This inconsistency could potentially create challenges for the regions’ planning and operations within healthcare (SNAO, 2014). Determining whether this is still the case should be a topic for future research, focusing on the regions’ perceptions of the agreements and the impact of the agreements on the regions’ discretion. Additionally, future research should explore other dimensions of the agreements as potential soft policy instruments, such as participation. It would be valuable to investigate to what extent the regions are involved in the process of negotiating and finalizing these agreements.

## Notes

1. Information is based on the authors’ calculations from compilations made by the Swedish Agency for Public Management between 2019 and 2022 (Statskontoret, 2020, 2021, 2022 and 2023). The numbers may vary depending on what is considered an agreement within the field of healthcare.

2. We have excluded three agreements in healthcare (Statskontoret, 2023, pp. 99–100): “The State’s Contribution to the Regions for Costs of Pharmaceutical Benefits etc.”, “Holistic, Equal, and Safe Care”, and “A Strategy for Implementing Vision for eHealth 2025: The Next Step”.

3. See: Link to the website

4. The only exception is disbursement in the agreement on cancer care, where half the amount is performance-based, and the other half is a standard amount (MoHSA/SALAR, 2021d).

## Appendix

**Table A1:** Agreements included in the empirical analysis

Cancer care (cancervård) “Equal and effective cancer care with shorter waiting times, 2022”, 26 pages (MoHSA/SALAR, 2021d)
Civil Defence (Civilt försvar) “The Healthcare’s Work with Civil Defence, 2022”, 22 pages (MoHSA/SALAR, 2021b) “The Healthcare’s Work with Civil Defence, 2022, Supplementary Agreement on Security of Supply for Medicines”, 5 pages
Covid-19 Testing (Covid-10 testning) “National Testing and Infection Tracing of Covid-19, 2022”, 8 pages (MoHSA/SALAR, 2021e)
Covid-19 Vaccination (Covid-19 vaccinering) “Implementing Vaccination against Covid-19, 2022”, 17 pages (MoHSA/SALAR, 2021a)
Increased Accessibility (Ökad tillgänglighet) “Increased Accessibility in Healthcare, 2022”, 26 pages (MoHSA/SALAR, 2022b) “Increased Accessibility in Healthcare, 2022, Supplement”, 7 pages
Integrated Care (God och nära vård) “Integrated Care, 2022. An Adjustment of the Healthcare with the Primary Care as the Hub”, 52 pages (MoHSA/SALAR, 2022a)
Mental Health (Psykisk hälsa) “Efforts in the Field of Mental Health and Suicide Prevention, 2021–2022”, 23 pages (MoHSA/SALAR, 2021c) “Efforts in the Field of Mental Health and Suicide Prevention, 2022, Supplement”, 5 pages
Women’s Health (Kvinnors hälsa) “Increased Accessibility and Equality in Maternal Health and Maternity Care and Strengthened Efforts for Women’s Health, 2020–2022”, 16 pages (MoHSA/SALAR, 2019) “Supplementary Agreement on Person-Centred, Accessible, and Equal Healthcare for Pregnancy, Childbirth and Aftercare, 2021–2022”, 16 pages “Amendment of Supplementary Agreement on Person-Centred, Accessible, and Equal Healthcare for Pregnancy, Childbirth and Post-Natal Care, 2021–2022”, 2 pages

**Note(s):** References refer to the main agreements. The number of pages does not include the title page, table of contents or appendices. Authors’ translation of titles

**Source(s):** Authors’ own work
